# Spatiotemporal Changes in Xylan-1/Xyloglucan and Xyloglucan Xyloglucosyl Transferase (XTH-Xet5) as a Step-In of Ultrastructural Cell Wall Remodelling in Potato–Potato Virus Y (PVY^NTN^) Hypersensitive and Susceptible Reaction

**DOI:** 10.3390/ijms19082287

**Published:** 2018-08-04

**Authors:** Katarzyna Otulak-Kozieł, Edmund Kozieł, Józef J. Bujarski

**Affiliations:** 1 Department of Botany, Faculty of Agriculture and Biology, Warsaw University of Life Sciences—SGGW, Nowoursynowska Street 159, 02-776 Warsaw, Poland; edmund_koziel@sggw.pl; 2 Department of Biological Sciences, Northern Illinois University, DeKalb, IL 60115, USA; jbujarski@niu.edu; 3 Institute of Bioorganic Chemistry, Polish Academy of Sciences, Noskowskiego 12/14, 61-704 Poznań, Poland

**Keywords:** cell wall, hypersensitive response, plant–virus interactions, Potato virus Y, immunolocalisation, ultrastructure, xyloglucan, xyloglucosyl transferase

## Abstract

One type of monitoring system in a plant cell is the cell wall, which intensively changes its structure during interaction with pathogen-stress factors. The wall plays a role as a dynamic and controlled structure, although it is not fully understood how relevant these modifications are to the molecular mechanisms during plant–virus interactions. In this work we localise the non-cellulosic polysaccharides such as xyloglucan, xylan (xylan-1) and xyloglucosyl transferase (XTH-Xet5), the enzyme that participates in the metabolism of xyloglucan. This provided us with information about the in situ distribution of the components of the hemicellulotic cell wall matrix in hypersensitive and susceptible potato–PVY^NTN^ interactions. The loosening of the cell wall was accompanied by an increase in xylan depositions during susceptible interactions, whereas, during the hypersensitive response, when the cell wall was reinforced, the xylan content decreased. Moreover, the PVY inoculation significantly redirected XTH-Xet5 depositions, regardless of types of interactions, compared to mock-inoculated tissues. Furthermore, the immunogold localisation clearly revealed the domination of Xet5 in the cell wall and in vesicles in the susceptible host. In contrast, in the resistant host increased levels of Xet5 were observed in cytoplasm, in the cell wall and in the trans-Golgi network. These findings show that the hypersensitive reaction activated XTH-Xet5 in the areas of xyloglucan endo-transglycosylase (XET) synthesis, which was then actively transported to cytoplasm, cell wall and to vacuoles. Our results provide novel insight into cell wall reorganisation during PVY^NTN^ infection as a response to biotic stress factors. These novel findings help us to understand the mechanisms of defence responses that are incorporated into the cell wall signalling network.

## 1. Introduction

Plant virus diseases are a major threat to crop production around the world. Viruses are widely known as a large, highly various but also economically important group of plant pathogens, and are the source of an enormous amount of pathological changes in plant tissues [1]. One of the most important plant viruses, Potato Y virus (PVY, genus Potyvirus, family *Potyviridae*), has been categorised as the fifth most economically damaging virus worldwide [2]. However, PVY is able to infect the wide spectrum of plant hosts from *Solanaceae* family, but also such ornamental plants like dahlia or petunia or some members of the families *Fabaceae* and *Chenopodiaceae*, as well as many other wild plant species [1]. Among the PVY strains, a whole variety of symptoms of infection are possible, from leaf crinkling to necrosis, and they hardly depend on the virus strain or the level of resistance. Although the recombinant strains like PVY^NTN^ or PVY^N-Wi^ usually induce mild or transient foliar mosaic symptoms, they are more likely to cause potato tuber necrotic ringspot disease (PTNRD) of varying severity in numerous potato cultivars. 

The plant host reactions to viral pathogens are mostly of two types, either molecularly compatible (susceptible) or incompatible (resistant) responses [3]. In the first interaction the virus potentially replicates and spreads systemically, whereas in the second, the systemic spread is frequently limited by the localised cell death, induced by the HR (hypersensitive response) [4,5]. In some experimental systems the virus might not be completely blocked [6]. Consequently, through the virus–host interactions, these pathogens remodel cellular membranes and organelles, hijack cellular pathways or engage diverse host factors to accomplish their life cycle [7,8]. Moreover, pathogens have evolved an “arsenal of tools” to explore their potential hosts. On the other hand, plants have evolved a variety of complex mechanisms leading to resistance, with the molecular monitoring systems able to trigger specific and/or adequate types of responses [9].

One element of plant monitoring system is the cell wall, which extensively changes its structure during interaction with a pathogen-stress factor. The wall functions as a dynamic and controlled structure, although the molecular mechanisms that control cell wall integrity are not fully understood [10], especially in view of plant–virus interactions. Previously, we suggested that the dynamic changes in the cell wall represent a key element of the potato defence network during interactions with PVY [11]. The analysis of CesA4, PR-2 and HRGP deposition within the plant apoplast and symplast confirmed their active trafficking as a step-in remodelling of the potato cell wall in response to PVY^NTN^ inoculation [11]. However, in many intensively studied plant–pathogen interactions the research on the remodelling of the cell wall has been largely restricted to non-viral pathogens [12,13]. Recent transcriptome and microarray studies on different plant–virus molecular dialogues clearly revealed that the cell-wall-related genes were subject to active up- or downregulation [14,15,16]. Therefore, in this study, we have analysed the non-cellulosic polysaccharides such as xylan-1 and xyloglucan xyloglucosyl transferase (XTH-Xet5), an enzyme involved in the metabolism of xyloglucan. We present our preliminary data about the localisation of the components of the hemicellulosic cell wall matrix during either compatible or incompatible PVY^NTN^–potato interactions. We observed changes in the distribution of selected hemicellulosic polysaccharides depending upon the PVY–potato interaction being hypersensitive or susceptible.

## 2. Results

### 2.1. Localisation of Xylan-1/Xyloglucan during Compatible and Incompatible PVY^NTN^–Potato Interactions

**Figure 1:**
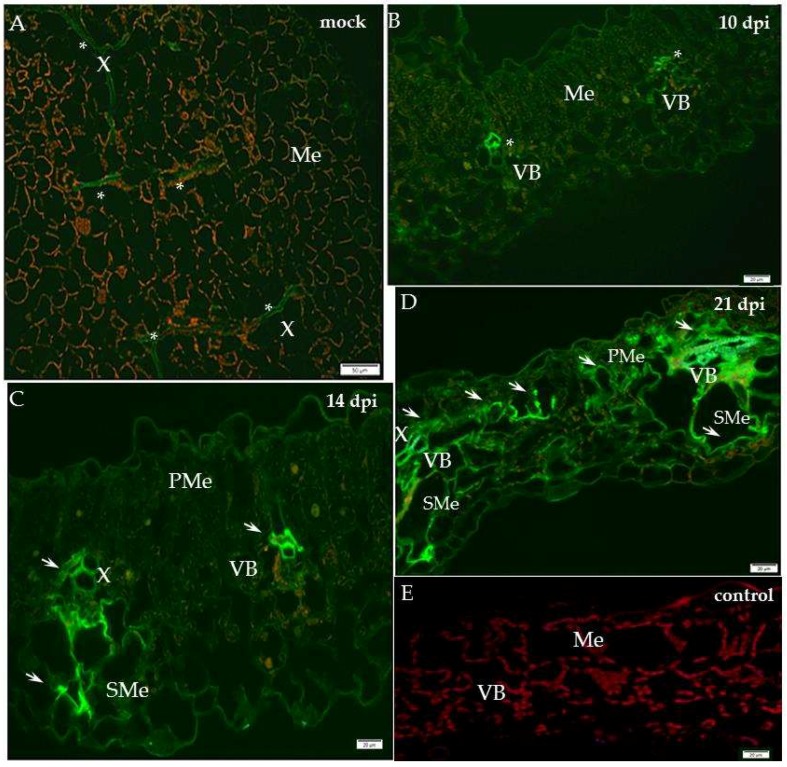
Immunofluorescence localisation of xylan-1/xyloglucan in potato–PVY^NTN^ compatible interaction. (**A**) Green fluorescence signal of xyl-1 in xylem tracheary elements (*) of mock-inoculated potato leaf on longitudinal section (control). Bar 50 µm. (**B**) Green fluorescence of xyloglucan in vascular bundle (VB, *) and in low intensity in mesophyll 10 days post-PVY^NTN^ inoculation. Bar 20 µm. (**C**) Fluorescence of xyl-1/xyloglucan in vascular bundle (arrows, phloem and xylem) and spongy mesophyll cells 14 days post-PVY^NTN^ inoculation. Lower intensity of signal in mesophyll. Bar 20 µm. (**D**) Strong signal of xyl-1/xyloglucan (arrows) in vascular bundle (phloem & xylem) of both types of mesophyll. Strong reorganisation of tissues’ patterns 21 days post-virus inoculation. Bar 20 µm. (**E**) Lack of green fluorescence signal when primary antibodies were omitted–control. Bar 20 µm. Me—mesophyll, PMe—palisade mesophyll, SMe—spongy mesophyll cell, VB—vascular bundle, X—xylem.

Our previous studies indicated the dynamic cell wall ultrastructural changes with variable levels of structural and remodelling cell wall proteins in the resistant and susceptible plants to PVY infection at 10 days after inoculation [11]. In the present research we studied the localisation of xylan-1 (CCRC-M108) and XTH-Xet5 (xyloglucan xyloglucosyl transferase) at four time points during PVY^NTN^–potato compatible and incompatible interactions. The immunofluorescence localisation of xylan-1/xyloglucan in susceptible potato leaves revealed that xyl-1 was induced during compatible PVY versus mock-inoculated plants (Figure 1). 

**Figure 2:**
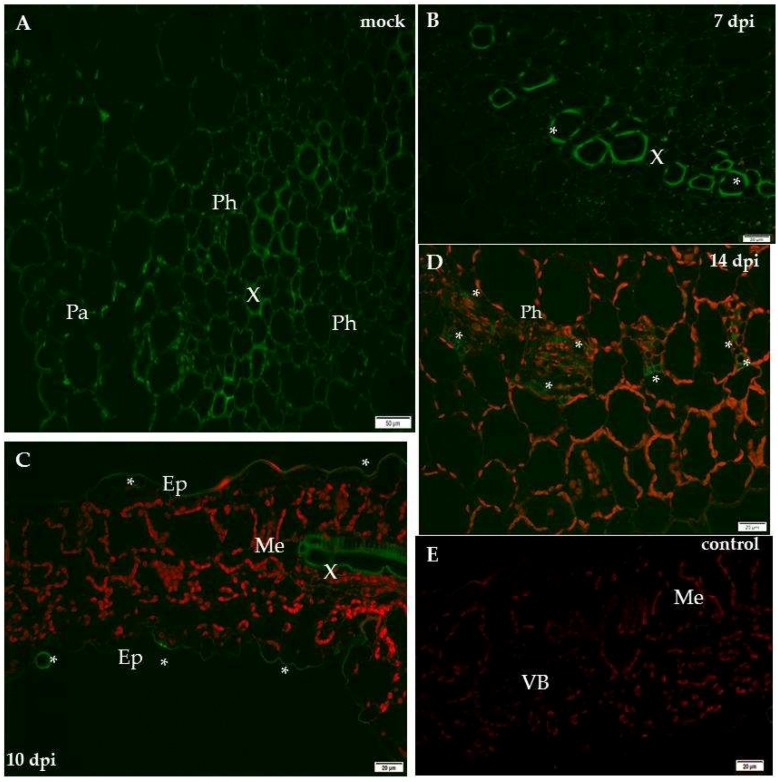
Immunofluorescence localisation of xylan-1/xyloglucan in potato–PVY^NTN^ incompatible interaction. (**A**) Green fluorescence signal of xyl-1 in xylem & phloem elements, also in parenchyma of mock-inoculated potato Sárpo Mira leaf. Bar 50 µm. (**B**) Green fluorescence signal of xyl-1 in xylem tracheary elements (*) 7 days post-PVY^NTN^ inoculation. Bar 20 µm. (**C**) Xyl-1/xyloglucan signal (*) in epidermis and xylem 10 days post-PVY^NTN^ inoculation. Bar 20 µm. (**D**) Xyl-1/xyloglucan signal (*) in phloem (*) 14 days post-PVY^NTN^ inoculation. Bar 20 µm. (**E**) Lack of green fluorescence signal when primary antibodies were omitted—control. Bar 20 µm. Ep—epidermis, Me—mesophyll, Pa—parenchyma cell, Ph—phloem, VB—vascular bundle, X—xylem.

**Figure 3:**
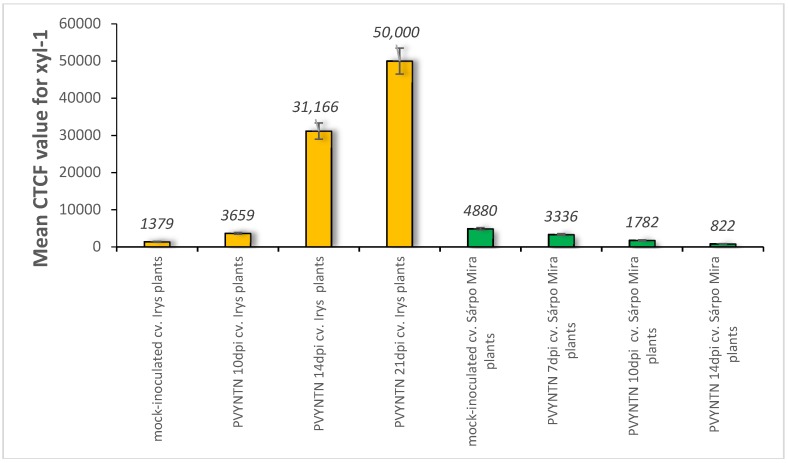
The assessment of the quantitative fluorescence signal of xylan-1/xyloglucan by using the corrected total cell fluorescence method (CTCF) with combination of ANOVA statistics analyses. Yellow bars indicate mock-inoculated and PVY-infected cv. Irys (susceptible) potato plants at 10, 14 and 21 days post-inoculation. Green bars represent mock-inoculated and PVY-inoculated Sárpo Mira (resistant) potato plants at 7, 10 and 14 days post-inoculation. Mean values CTCFC were evaluated at the *p* < 0.05 level of significance using post-hoc Tukey HSD test.

In contrast, in the hypersensitive reaction the level of xyl-1 deposition decreased (Figure 2). In the susceptible cultivar Irys, the green fluorescence signal was observed mainly in the phloem and at lower intensity also in the mesophyll, 10 days past virus inoculation (dpi), after the symptoms appeared (Figure 1B). Unlike in mock-inoculated Irys, xylan-1 was detected only in xylem tracheary elements (Figure 1A). During our investigation we analysed the localisation of xylan1/xyloglucan at all time points after mock inoculation—in hypersensitive potato Sárpo Mira at 7, 10 and 14 days post-mock-inoculation and for sensitive potato at 10, 14, 21 day post-mock-inoculation—to check the development of potential influence on xyl-1 deposition. However, no significant differences in localisation of xylan1/xyloglucan were noticed between time points in all mock-inoculated potato plants. The lack of the green fluorescence signal was observed in the potato tissue after incubation with pre-immune serum as well as when primary antibodies were omitted (Figure 1E and Figure 2E). Fourteen days after PVY inoculation, the strongest signal was detected in xylem, but also in spongy mesophyll cells (Figure 1C). After 21 days the xyl-1/xyloglucan signal was observed in both the vascular tissues and in the two types of mesophyll, alongside the tissue reorganisation as a consequence of virus infection (Figure 1D). The quantitative measuring of the fluorescence confirmed a statistically significant increase of xyl-1 signal in susceptible potatoes at all time points dpi, alongside a decrease in xyl-1 during the hypersensitive response compared to mock-inoculated plants (Figure 3). Moreover, as the infection progressed in the compatible interaction, the deposition of xylan-1 increased (Figure 1 and Figure 3). On the contrary, in potato Sárpo Mira the xyl-1 signal gradually reduced starting at seven dpi, when the reaction symptoms developed (Figure 2 and Figure 3). Seven dpi xyl-1 was found mainly in xylem cells (Figure 2B), whereas after 10 days a weak signal also appeared in the epidermis (Figure 2C), and the lowest signal was observed in phloem cells 14 days after inoculation (Figure 2D). 

**Figure 4:**
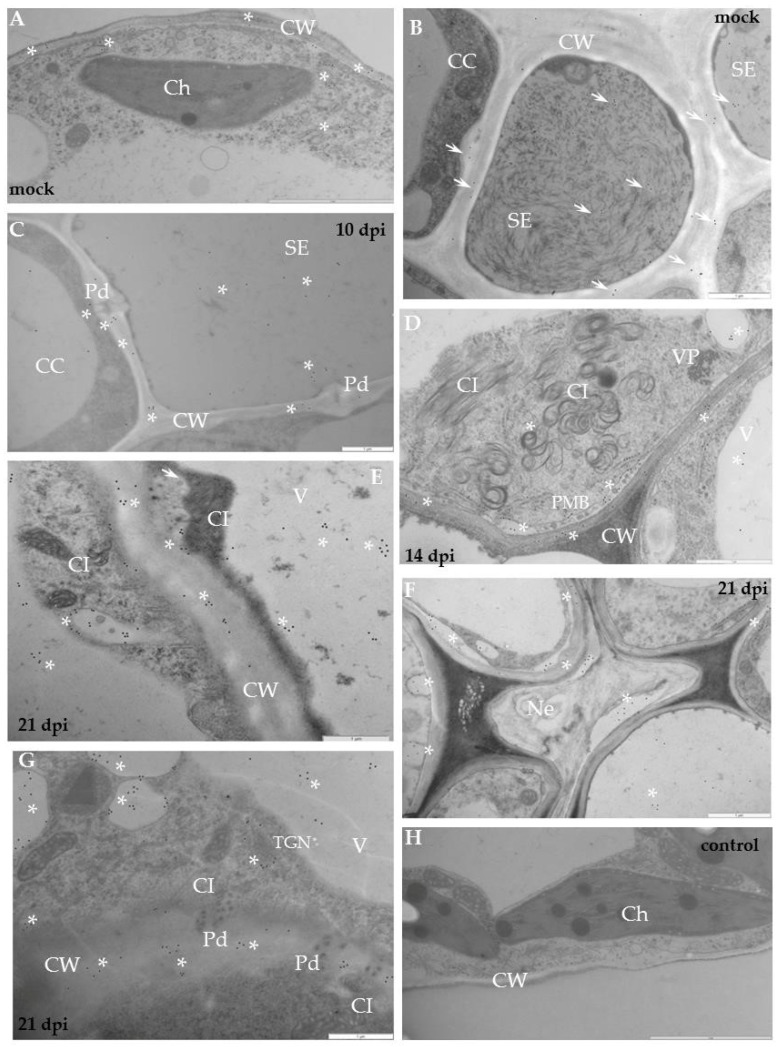
Immunogold labelling of xylan-1/xyloglucan for potato–PVY^NTN^ compatible interaction. (**A**) Gold deposition (*) of xyl-1 in cell wall and attached to vesicular and membranous structures in mesophyll cells of mock-inoculated leaf. Bar 2 µm. (**B**) Xylan-1/xyloglucan localisation (arrows) in cell wall and inside sieve elements in phloem of mock-inoculated tissue. Bar 1 µm. (**C**) Xylan-1/xyloglucan localisation (*) in cell wall around plasmodesmata and inside sieve elements in the phloem 10 days post-PVY^NTN^ inoculation. Bar 1 µm. (**D**) Gold deposition (*) of xyl-1 in the area between cell wall and plasmalemma with paramular bodies, in vacuoles and in cell wall of mesophyll cell where virus particles (VP) and inclusions (CI) were deposited 14 days post-inoculation. Bar 1 µm. (**E**) Xylan-1/xyloglucan localisation (*) in cell wall, vesicles and vacuole in mesophyll cell next to virus cytoplasmic inclusions. Plasma membrane retractions (arrow) from cell wall. Bar 1 µm. (**F**) Xylan-1/xyloglucan (*) deposited in the area undergoing necrotisation 21 days post-inoculation Bar 1 µm. (**G**) Xylan-1/xyloglucan (*) deposited in cell wall around plasmodesmata and in vacuoles 21 days post-inoculation. Virus inclusions (CI) are visible next to plasmodesmata. Bar 1 µm. (**H**) Lack of gold deposition during potato Irys–PVY^NTN^ compatible interaction when primary antibodies were omitted (control). Bar 2 µm. CC—companion cell, Ch—chloroplast, CI—cytoplasmic inclusions, CW—cell wall, Ne—necrosis, Pd—plasmodesmata, PMB—paramular bodies, SE—sieve element, V—vacuole, VP—virus particles.

Immunogold labelling revealed that in mock-inoculated potato Irys the xyl-1 epitopes appeared in the endoplasmic reticulum and in the trans-Golgi network or in other vesicular and membranous structures (Figure 4A,B). In compatible PVY–Irys interaction, xyl-1 appeared in the cell wall around plasmodesmata in the phloem tissue (Figure 4C), as well as in the mesophyll (Figure 4E,G). Gold deposition was often associated with vacuoles (Figure 4C–G) and vesicular structures (Figure 4E,F), also with paramular bodies between cell wall and plasmalemma (Figure 4D). Additionally, xyl-1 epitopes were deposited in the area undergoing necrotisation 21 dpi, especially alongside PVY particles or inclusion bodies (Figure 4F). 

Quantification by immunogold of xylan epitopes revealed an increase of xyl-1 in potato Irys infected with PVY^NTN^ (Table 1). Moreover, during compatible interaction a statistically significant amount of xylan was detected in the cell wall, in vacuoles containing vesicles, as well as in the trans-Golgi network and the endoplasmic reticulum. In all these compartments the deposition was much higher than in mock-inoculated Irys plants. The above results clearly indicate that xylan/xyloglucan was activated as a result of compatible potato–PVY interaction, but also that its distribution and deposition were visibly changed compared to healthy plants. 

**Figure 5:**
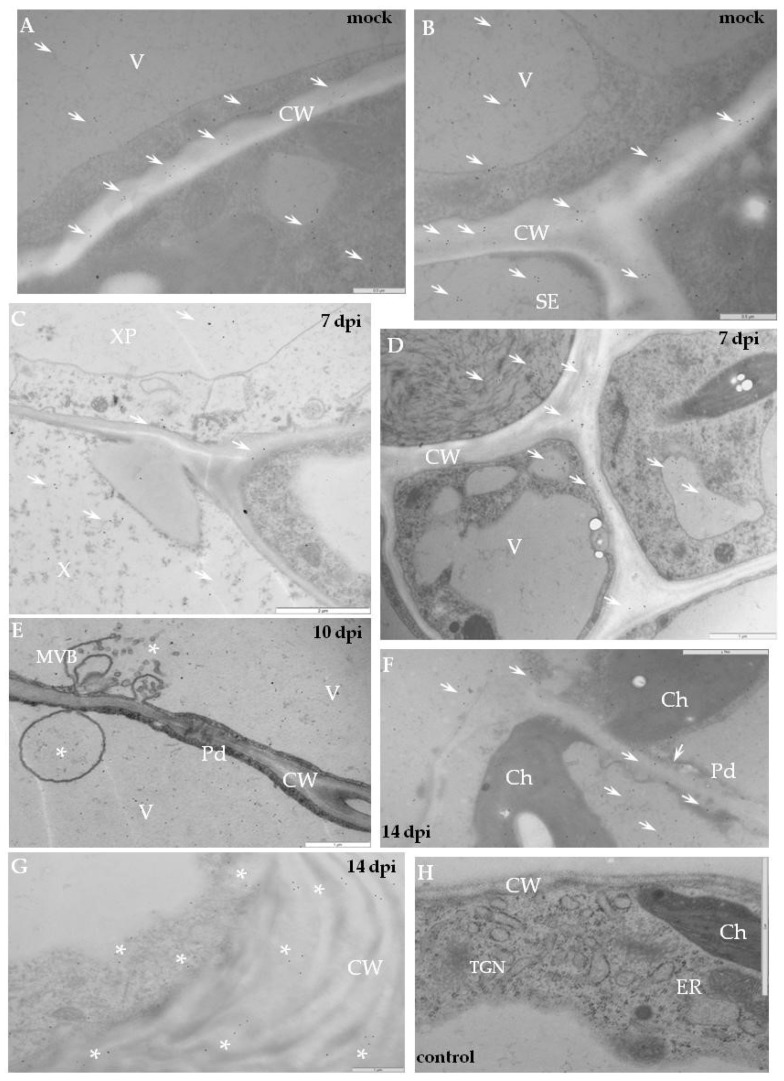
Immunogold labelling of xylan-1/xyloglucan for potato–PVY^NTN^ incompatible interaction. (**A**) Xylan-1/xyloglucan localisation (arrows) in cell wall, vacuole and in cytoplasm, also associated with membranes. Bar 0.5 µm (**B**) Xylan-1/xyloglucan localisation (arrows) in companion cells and sieve element of mock-inoculated potato Sárpo Mira leaf. Depositions are visible in the cell wall, vacuole and cytoplasm. Bar 0.5 µm (**C**) Xylan-1/xyloglucan deposition (arrow) in xylem tracheary element and xylem parenchyma seven days post-PVY inoculation. Bar 2 µm (**D**) Xylan-1/xyloglucan localisation (arrows) inside sieve element, also in cell wall and vacuole of phloem parenchyma cells seven days post-PVY^NTN^ inoculation. Bar 1 µm (**E**) Deposition in vacuole with multivesicular bodies (*, MVB) 10 days past virus inoculation. Bar 1 µm (**F**) Xyloglucan localisation (arrows) closely related with cell wall plasmodesmata 14 days after PVY inoculation in palisade mesophyll cell. Bar 1 µm (**G**) Xyl-1 deposition (*) in cell wall and cytoplasm in collenchyma of potato Sárpo Mira leaf 14 days post virus inoculation. Bar 1 µm. (**H**) Lack of gold deposition in mesophyll of potato Sárpo Mira leaf–PVY^NTN^ compatible interaction when primary antibodies were omitted (control). Bar 2 µm. Ch—chloroplast, CW—cell wall, MVB—multivesicular bodies, Pd—plasmodesmata, SE—sieve element, TGN—trans-Golgi network, X—xylem tracheary element, XP—xylem parenchyma.

The immunogold localisation of xylan in the hypersensitive potato Sárpo Mira confirmed the above fluorescence analyses. The gold deposition in mock-inoculated resistant plants was higher than in susceptible potatoes, similar to the fluorescence data (Figure 4A,B and Figure 5A,B, Table 1). In healthy Sárpo Mira tissues the xyl-1 was detected mainly in the cell wall and in vacuoles (Figure 5A,B). When the symptoms of hypersensitive response to PVY were visible, the xylan was observed in the vascular tissues, in xylem tracheary elements with xylem parenchyma as well as in phloem sieve elements (Figure 5C,D). After hypersensitive response, xylan was noticed in mesophyll tissue close to the plasmodesmata (Figure 5E,F), but also associated with multivesicular bodies (Figure 5E). Additionally, xylan accumulated in the collenchyma at lower intensity than in vascular bundles or the mesophyll (Figure 5G). Regardless of the type of interaction, a lack of gold depositions was noticed in those tissue sections that were incubated with pre-immune serum or when primary antibodies were omitted (Figure 4H and Figure 5H). 

Quantification of xylan-1 antigen by immunogold in the host cell compartments revealed a statistically significant decrease in xyloglucan deposition during hypersensitive reaction, compared to controls (Figure 5, Table 1). As an effect of virus infection, the xyl-1 epitope reached the highest level in the cytoplasm and in vacuoles but was at a lower level in the cell wall and in the trans-Golgi network, whereas, in the control mock-inoculated plants the depositions took place mainly in vacuoles and in the cell wall. 

Moreover, the distribution of xyl-1/xyloglucan was generally different after compatible as opposed to incompatible interactions. The content of xyl-1 in susceptible potatoes was induced by PVY (Figure 1, Figure 3 and Figure 4, Table 1). On the contrary, in hypersensitive potato the level of xyl-1 decreased (Figure 2, Figure 3 and Figure 5, Table 1). Also, after compatible interaction the most intense deposition occurred in cell walls and in vacuoles, and was associated with the membranous compartments such as ER, trans-Golgi network or vesicular structures (Figure 4, Table 1), whereas in hypersensitive reaction the highest level of xyloglucan was found in the cytoplasm and in vesicular structures (Figure 5, Table 1). These data clearly indicate that virus infection can cause varied xylan-1 distribution in potato tissues, depending upon the level of anti-PVY^NTN^ resistance.

### 2.2. Localisation of Xyloglucan Xyloglucosyl Transferase (XTH-Xet5) (E.C. 2.4.1.207) during Compatible and Incompatible PVY^NTN^–Potato Interactions

**Figure 6:**
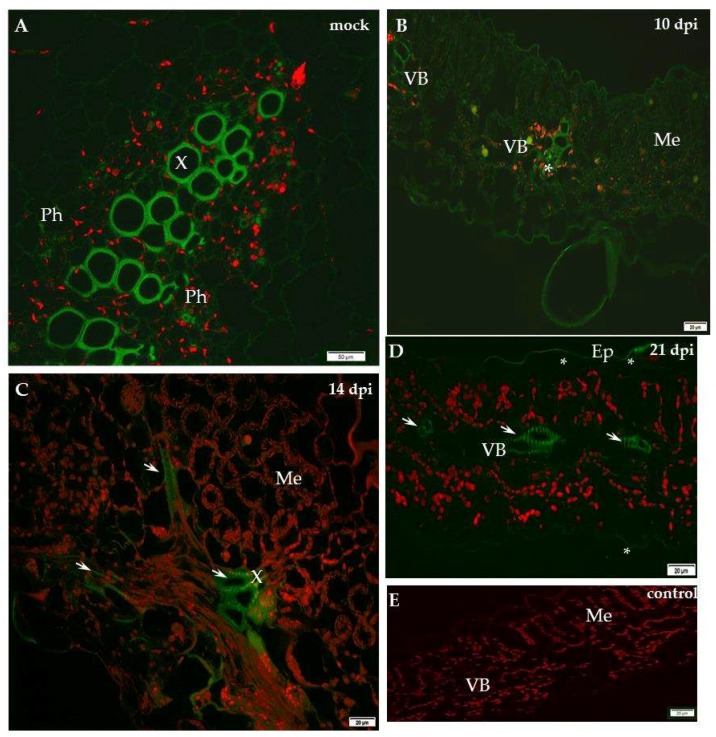
Immunofluorescence localisation of xyloglucan xyloglucosyl-transferase (XTH-Xet5) during potato–PVY^NTN^ compatible interaction. (**A**) Green fluorescence signal of XTH-Xet5 in xylem and phloem elements of mock-inoculated potato Irys leaf petiole. Bar 50 µm. (**B**) Fluorescence detection of XTH-Xet5 in xylem, phloem parenchyma (*) and weaker signal in the mesophyll of Irys leaflets 10 days post-virus inoculation. Bar 20 µm (**C**) Fluorescence detection of XTH-Xet5 in xylem (arrows) 14 days post-PVY inoculation. Bar 20 µm (**D**) Detection of XTH-Xet5 in xylem (arrows) and epidermis (*) 21 days post-PVY inoculation. Bar 20 µm (**E**) Lack of green fluorescence signal when primary antibodies were omitted–control. Bar 20 µm. Ep—epidermis, Me—mesophyll, Ph—phloem, VB—vascular bundle, X—xylem.

For compatible PVY–potato (cv. Irys) interaction, a strong green fluorescence signal of XTH-Xet5 was observed predominantly in the vascular bundle in mock-inoculated plants, whereas a weaker signal was seen from mesophyll cells 10 days post-inoculation (Figure 6A,B). The localisation of XTH/Xet5 at all time points after mock-inoculation—in hypersensitive potato Sárpo Mira at 7, 10 and 14 days post-mock-inoculation and for sensitive potato at 10, 14, 21 days post-mock-inoculation—were analysed to check the potential developmental influence on XTHE/Xet5 deposition. However, there were no significant differences in the localisation of Xet5 between time points of all mock-inoculated potato plants. Fourteen dpi a XTH-Xet5 signal was detectable almost exclusively in xylem tracheary elements, similar to 21 days post-inoculation, where besides xylem cells the green fluorescence was noticed in the epidermis outer cell wall (Figure 6 C,D). 

**Figure 7:**
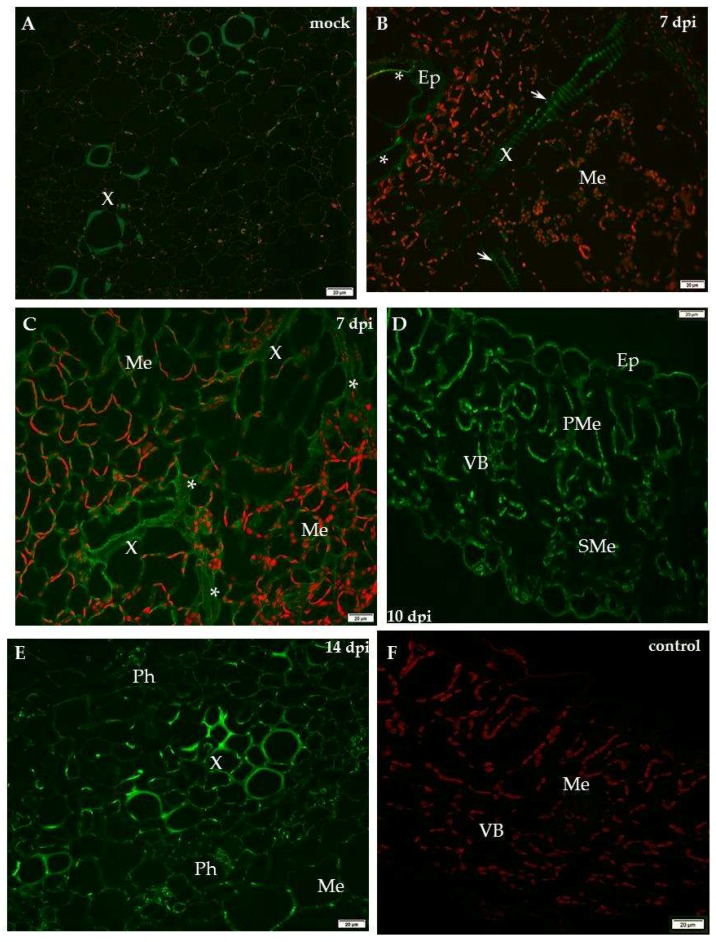
Immunofluorescence localisation of xyloglucan xyloglucosyl-transferase (XTH-Xet5) during potato–PVY^NTN^ incompatible interaction. (**A**) Green fluorescence signal of XTH-Xet5 in xylem tracheary elements of mock-inoculated potato Sárpo Mira leaf base. Bar 50 µm. (**B**) XTH-Xet5 detection in xylem (arrows) and epidermis (*) 7 days post-PVY^NTN^ inoculation. Bar 20 µm (**C**) Fluorescence detection of XTH-Xet5 in xylem, phloem (*) and mesophyll 7 days post-PVY^NTN^ inoculation. Bar 20 µm (**D**) Fluorescence detection of XTH-Xet5 in all leaflet tissues 10 days post-PVY inoculation. Bar 20 µm. (**E**) XTH-Xet5 green fluorescence signal in xylem & phloem and mesophyll in Sárpo Mira leaf base 14 days post-PVY^NTN^ inoculation. Bar 20 µm. (**F**) Lack of green fluorescence signal when primary antibodies were omitted—control. Bar 20 µm. Ep—epidermis, Me—mesophyll, Ph—phloem, PMe—palisade mesophyll, SMe—spongy mesophyll cell, VB—vascular bundle, X—xylem.

**Figure 8:**
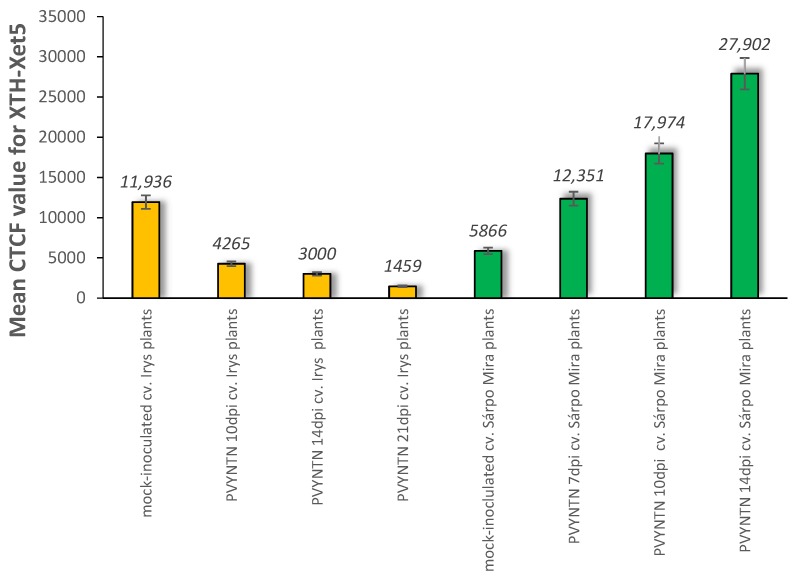
The assessment of the quantitative fluorescence signal of XTH/Xet5 by using the corrected total cell fluorescence method (CTCF) in combination with ANOVA statistical analyses. Yellow bars indicate mock-inoculated and also PVY inoculated cv. Irys (susceptible) potato plants at 10, 14 and 21 days post-inoculation. Green bars indicate mock-inoculated and also PVY-inoculated Sárpo Mira (resistant) potato plants at 7, 10 and 14 days post-inoculation. Mean values CTCFC were evaluated at the *p* < 0.05 level of significance using post hoc Tukey HSD test.

The immunofluorescence analyses of the hypersensitive reaction tissue provided a significantly different picture. In mock-inoculated hypersensitive cv. Sárpo Mira, the XTH-Xet5 antigen was detected only in the cell wall of xylem tracheary elements, a different pattern than in cv. Irys (Figure 7A). Seven days after PVY inoculation of Sárpo Mira, the green fluorescence signals were detected not only in both xylem and in phloem tissue, but also in the cell wall of mesophyll cells (Figure 7B,C). Moreover, starting from 10 days post-inoculation the XTH-Xet5 antigen signals were noticed in all leaf and petiole cells, the most intensely visible in both vascular tissues (Figure 7D,E). The fluorescence signal was not detectable in tissue sections incubated with the pre-immune serum or when primary antibodies were omitted (Figure 6E and Figure 7F). The immunofluorescence-based quantitative measurement of total cell fluorescence (CTCF) clearly indicated the statistically significant stepwise increase of the XTH-Xet5 antigen signal during hypersensitive response. To the contrary, during susceptible potato–PVY interaction the XTH-Xet5 signal gradually decreased (Figure 8).

**Figure 9:**
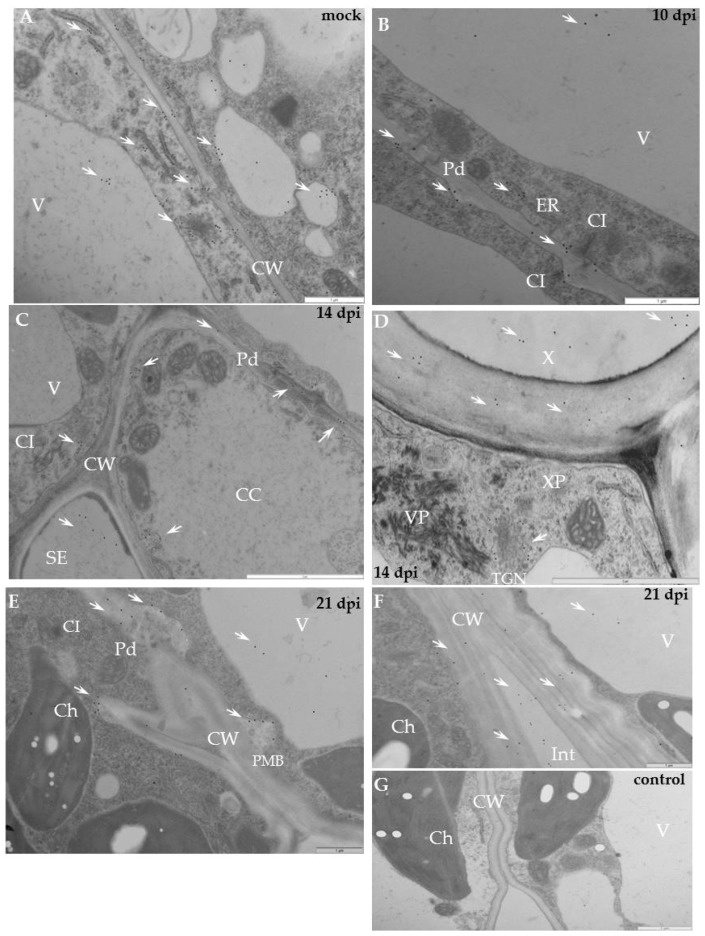
Immunogold labelling of xyloglucan xyloglucosyl-transferase (XTH-Xet5) during potato–PVY^NTN^ compatible interaction. (**A**) Gold granules (arrows) of XTH-Xet5 deposition are visible in cell wall, vacuoles as well as around ER and trans-Golgi network in mesophyll cells of mock-inoculated potato Irys leaf. Bar 1 µm (**B**) XTH-Xet5 deposition (arrows) in cell wall, around plasmodesmata and in vacuole. Virus cytoplasmic inclusions next to plasmodesmata 10 days post-inoculation. Bar 1 µm (**C**) XTH-Xet5 localisation (arrows) in sieve element, also in cell wall next to plasmodesmata in phloem parenchyma 14 days post-virus inoculation. Bar 2 µm. (**D**) XTH-Xet5 (arrows) in xylem tracheary element and around the trans-Golgi network. Viral particles are noticeable in xylem parenchyma cell. Bar 2 µm. (**E**) XTH-Xet5 depositions (arrows) are in loosened cell wall, around plasmodesmata and in vacuole. Plasma membrane retraction from cell wall with paramular bodies. Virus inclusions are in cytoplasm 21 days post-virus inoculation. Bar 1 µm. (**F**) XTH-Xet5 deposition (arrows) in cell wall and in intercellular space 21 days post-virus inoculation. Bar 1 µm. (**G**) Lack of gold deposition for potato Irys–PVY^NTN^ compatible interaction when primary antibodies were omitted (control). Bar 2 µm. CC—companion cell, Ch—chloroplast, CI—cytoplasmic inclusions, CW—cell wall, Pd—plasmodesmata, SE—sieve element, TGN—trans-Golgi network, V—vacuole, VP—virus particles, X—xylem, XP—xylem parenchyma.

**Figure 10:**
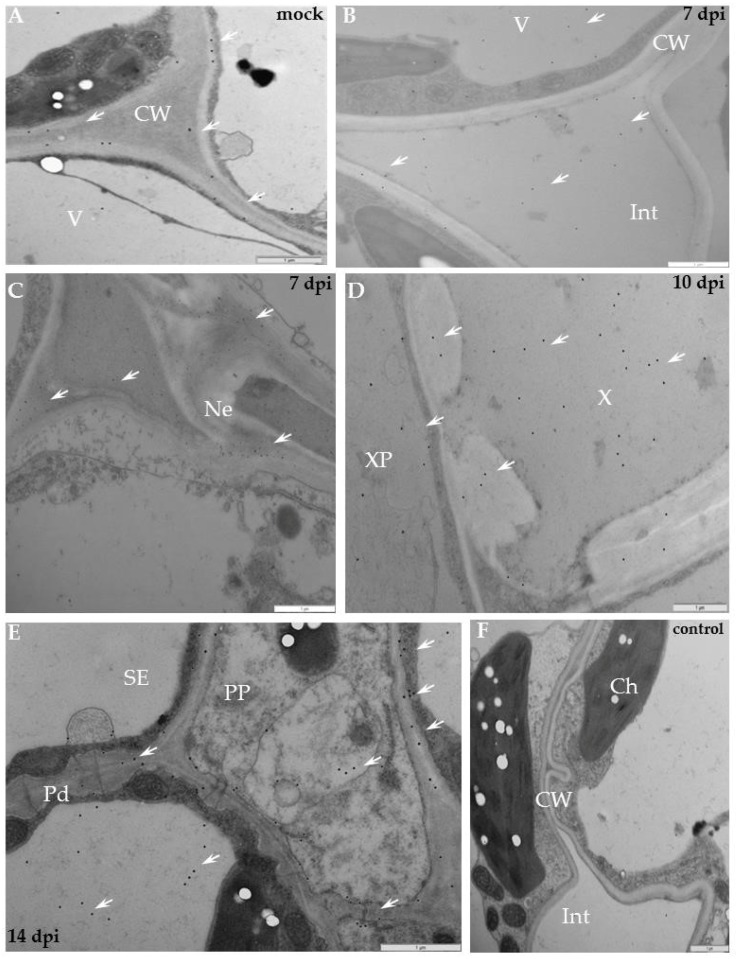
Immunogold labeling of xyloglucan xyloglucosyl-transferase (XTH-Xet5) after potato–PVY^NTN^ incompatible interaction. (**A**) Gold granules of XTH-Xet5 depositions (arrows) in cell wall and in vacuoles of mock-inoculated Sárpo Mira plant. Bar 1 µm. (**B**) Gold granules of XTH-Xet5 deposition (arrows) present in cell wall, intercellular space, cytoplasm and in vacuole seven days post-inoculation. Bar 1 µm. (**C**) XTH-Xet5 localisation (arrows) in cell wall and in necrotised area seven days post-inoculation. Bar 1 µm. (**D**) Deposition (arrow) present in xylem tracheary element and in xylem parenchyma cell 10 days post-PVY inoculation. Bar 1 µm. (**E**) XTH-Xet5 deposition (arrows) in cell wall, vesicular/membranous structures and in plasmodesmata rounded areas in phloem, 14 days post-inoculation. Bar 1 µm. (**F**) Lack of gold depositions in potato Sárpo Mira–PVY^NTN^ compatible interaction when primary antibodies were omitted (control). Bar 1 µm. Ch—chloroplast, CW—cell wall, Int—intercellular space, Ne—necrosis, Pd—plasmodesmata, PP—phloem parenchyma, SE—sieve element, V—vacuole, X—xylem, XP—xylem parenchyma.

In cv. Irys, the XTH-Xet5 antigen was detected by immunogold labelling mainly in cell wall, along endoplasmic reticulum and the vacuoles (Figure 9A). As a result of PVY inoculation, the XTH-Xet5 antigen was deposited firstly in a loosened cell wall around plasmodesmata alongside the virus inclusions, and thereafter in vesicular structures (Figure 9B,C,E). In vascular tissues the gold was mainly associated with sieve elements, phloem parenchyma as well as with xylem tracheary elements and xylem parenchyma (Figure 9C,D), especially when virus inclusions and particles were present. Moreover, gold granules were found not only in the cell wall or along the plasmalemma, but even in the intercellular space (Figure 9F). In resistant potato Sárpo Mira, the transferase epitopes were detected mostly in the cell wall area, but also in vacuoles (Figure 10A). After hypersensitive response to PVY infection, similar to compatible interaction, the presence of XTH-Xet5 antigen localised to the cell wall, between plasma membranes and the cell wall of the necrotised area, or even in the intercellular space (Figure 10B,C,E). In the phloem the preferable places of gold deposition were vesicular/membranous structures and the plasmodesmata rounded areas (Figure 10E). In xylem the depositions were noticed inside xylem tracheary elements and in xylem parenchyma (Figure 10D). The gold granules were absent in control sections (Figure 9G and Figure 10F). The immunogold quantification clearly demonstrated a significantly higher deposition of XTH-Xet5 after hypersensitive response to PVY infection than after the compatible interaction (Figure 9 and Figure 10, Table 1). Interestingly, the xyloglucan transferase protein reached much higher levels of XTH in mock-inoculated Irys potato than in resistant Sárpo Mira. A statistically significant deposition of gold granules in mock-inoculated Sárpo Mira was detected only in the cell wall and in vacuoles. After PVY^NTN^ inoculation, the highest level of XTH-Xet5 in Sárpo Mira was in the cytoplasm, cell wall and trans-Golgi network, whereas in Irys the XTH-Xet5 was found in vesicular structures and in the cell wall (Table 1). 

Moreover, the deposition of XTH-Xet5 epitopes after compatible interaction was significantly lower, compared to mock-inoculated plants. During hypersensitive response, XTH-Xet5 dominated in cytoplasm at almost the same level as in the cell wall and around the trans-Golgi network. This is unlike in the susceptible host Irys, where transferase was mainly deposited in the cell wall and in vesicular structures, but at a much lower level then in HR. Moreover, the location in the cytoplasm in Irys was at the lowest level. The quantification statistics implied the PVY infection to modify the distribution of xyloglucan transferase in the tissues and cell compartments of Sárpo Mira and Irys plants, depending on the type of virus–host interactions.

## 3. Discussion

The cell wall components contribute to plant growth, development and cell interaction with different stimuli such as the cascade of signal transduction factors, both biotic and abiotic [17]. The cell wall acts as a first line of defence. The composition of the cell wall can serve as a molecular signature of the environmental monitoring as many components undergo synthesis and/or hydrolysis during stress [9]. Therefore, the function of different cell wall components and the question of how they interact with each other and with exogenous factors (such as pathogens) have been a subject of extensive research for many years [18,19]. 

Some of these questions may be answered based on the first published transcriptome dataset of the response to different groups of pathogens [18,19,20]. These analyses revealed the effects of plant–virus interactions [14,21], especially about the reinforcing of the cell wall structure. Our knowledge of the mechanisms of resistance to plant pathogens was extended by RNAseq analyses of the whole transcriptomes [14]. In general, the expression of genes that coded for key protein/enzymes participating in the synthesis of cell wall components can be significantly repressed by virus infection [22,23]. 

More recently, it has been reported that expression of genes modifying cell wall could be actively enhanced, as response to the stress-related cell wall signalling [21,24]. The current view is that the plant cell wall forms a functional network that is able to resist via a dynamic extracellular complex of polysaccharides, together with glycoproteins and the modifying enzymes [25]. Moreover, the metabolism of cell wall polysaccharides can regulate the balance between biosynthesis and degradation, and the shift of this balance can lead to structural changes in the cell wall [25]. 

The unique composition of hemicellulosic polysaccharides, including xyloglucans, xylans and the polysaccharide-modifying enzymes, can be modified due to the response to the pathogen-related stress. Bacete et al. [9] demonstrated that the metabolism of xyloglucan plays an important role in the expansion of cell wall, affecting the pathogen invasion. The microbial pathogens not only break the cell wall, but the β-1,4 xylanase is induced to degrade xylans producing endoxylanases, and this mechanism is also relevant to viral infections [26]. Recent microarray analysis and the transcriptome data demonstrate the regulation of xyloglucan metabolism during different types of plant–virus interactions [14,27]. Indeed, xylan biosynthesis as well as the metabolism of glucuroxylan seem to represent the most common down-regulated functional categories after the transcriptomics analyses of the *Tobacco etch virus* (Potyvirus, TEV) infected *Arabidopsis* plants [27]. *Rice stripe virus* (RSV) suppressed the hypersensitive reaction in rice resistant varieties, with downregulated pathogenesis-related proteins, whereas changes in the xyloglucan endo transglycosylase/hydrolase could lead to cell wall strengthening, conditioning the resistance mechanism to RSV in rice [14]. 

In this work we analysed the effects of *Potyvirus* infection on selected hemicelluloses, non-cellulosic cell wall polysaccharides in both susceptible and resistant potato hosts. Indeed some papers mentioned above concentrated on gene regulation and transcriptomes being triggered by host–virus interactions. To gather complex information about the role of xylan/xyloglucan in cell wall integrity during virus infection, here we have analysed in situ localisation and the distribution of selected components of xyloglucan metabolism such as xylan-1/xyloglucan and XTH-Xet5 after compatible and hypersensitive responses during PVY^NTN^–potato infection. 

Our findings reveal that xyl-1/xyloglucan was induced after PVY^NTN^-infection in susceptible potato cv. Irys. Moreover, this tendency correlated closely with the appearance of the necrotic symptoms, being fully developed 21 days after virus inoculation. Unexpectedly, the observed dynamic changes in deposition of the major non-cellulosic polymer correlated well with the observed distribution of xyloglucans after reconstruction of the cell wall during the formation of syncytia, induced by nematodes [28]. The most intense deposition of xylan/xyloglucan was in vascular bundles (xylem and phloem) and in mesophyll, but also in the virus-induced necrotising areas. Similar data were reported by Northcote et al. [29], detecting xylan in the growing cell wall and in the xylem differentiating and thickening cell walls plus in mesophyll cells. Along these lines Northcote et al. [29] observed gold location in the cell wall, and a relatively similar intensity in and around vesicles, as well as in the trans-Golgi networks. 

Xyloglucan can be found in almost every plant species and is the most abundant hemicellulose of the primary wall [30]; it is closely linked to pathogen-induced changes. Our observations confirmed that cell wall loosening was accompanied by an increase in xylan deposition in the PVY-infected potato Irys. These results are also linked to the increase of viral presence measured by ELISA (Table S1). In contrast, during the hypersensitive response, the cell wall was reinforced, while the xylan content decreased, similar to the interactions of nematodes on potatoes or on *Arabidopsis*. The latter has roots deprived of the secondary cell wall and thus does not carry xylans [31]. Moreover, our findings clearly indicate that the distribution of xyl-1/xyloglucan depends upon the types of reaction to the PVY^NTN^ infection. That is, after compatible interaction the xylan epitope dominated in cell wall and in vacuoles, whereas after hypersensitive response the epitope was redistributed mainly to the cytoplasm and to vesicles.

The plant cell wall contains numerous enzymes that modify polysaccharides [32], and xyloglucan endotransglycosylase/hydrolase (XET, XTH) is an essential constituent, especially in the primary cell wall, participating in wall construction and elongation [33]. XET cleaves the xyloglucan chain endolytically and forms a covalent polysaccharide–enzyme complex. XTH/Xet is commonly thought to participate in cell wall loosening during plant development and wall expansion as well as increase rigidity during pathogen intrusion [34]. Transgenic *Arabidopsis* expressing *Capsicum annuum* XTH revealed distorted leaves, carrying irregular cell pattern in cross sections [35]. Further analysis suggested the role of XTH in cell wall remodelling. *Zea mays* XET1 is likely involved in the wall extension via hydrolysis and rejoining the xyloglucan molecules [36]. Additionally, xyloglucan holds seven glucosidic linkages and their formation requires different enzymes, including a complex of one xyloglucansynthase and fucosyltransferase, two galactosyltransferases and three xylosyltransferases [37]. 

Our analyses of compatible and incompatible interactions of PVY with potato cultivars focused on the deposition of xyloglucan xylosyl transferase (XTH-Xet5). Based on seven *Arabidopsis* genes encoding the xyloglucan xylosyltransferase, which are not fully characterised [38], XTH-Xet5 allegedly belongs to group GH16 of glycoside hydrolases. Moreover, Xet5 catalyses in vitro the formation of covalent linkages between xyloglucans and cellulosic substrates or even between xyloglucan and (1,3-and 1,4-) d-glucan [39,40]. It is thus possible that XTH-Xet5 is responsible for the linking of different polysaccharides in vivo, which consequently affects the strength of cell walls, but also decreases their flexibility and porosity. Our data follow these general considerations. Just as we observed the most intense XTH-Xet5 deposition during hypersensitive reaction, it has also been observed during the strengthening of cell walls in the cv. Sárpo Mira [11]. The analysis of cell wall metabolism during infection of papaya with PMeV (*Papaya meleira virus*) showed that this interaction upregulated xyloglucan endotransglycosylase activity [41]. Also, similar to our observations and to the data on PMeV infection, xyloglucan endotransglycosylases were affected by Potato leafroll virus (PLRV) infection in potatoes [42]. Moreover, higher deposition of XTH-Xet5 was correlated with a decrease in the virus presence measured by ELISA (Table S1).

The results from two other systems are also relevant to PVY^NTN^ interaction with susceptible and resistant potatoes. Namely, in the susceptible *A. thaliana*–*Turnip mosaic virus* (TuMV, Potyvirus) system the dramatic downregulation of XTH6 was observed in the wild-type plant or even in a defective silencing mutant, with both the qRT-PCR and the microarray assays [43]. Our results on the lowered level of XTH-Xet5 in susceptible potatoes were also observed in *Arabidopsis*, compared to mock-inoculated plants. As in Rose et al. [34], the transfer of xyloglucan was observed, also shifting the function of XTH toward the breakdown of the xyloglucan–cellulose network [43]. Our data from the PVY^NTN^–Sárpo Mira hypersensitive interaction also suggest the potential involvement of XTH in the hydrolysis of the xyloglucan–cellulose network. We have shown that the higher activity of XTH-Xet5 paralleled the lower level of xyl-1/xyloglucan. We have also previously observed a lowered level of CesA4 during HR than for susceptible interaction. 

Secondly, the five plant genes involved in cell wall metabolism were identified to operate during PVY infection in the resistant VAM seedlings of *Nicotiana tabacum* [44]. The genes associated with the cell wall structure were downregulated, while those participating in the remodelling tend to be upregulated. On the contrary, the identified xyloglucan endotransglucosylase hydrolase (JZ 897688) was downregulated at 12 h post-inoculation, but upregulated starting from day one after infection [44]. Our findings, similar to Chen et al.’s [44], reveal a gradually increasing level of XTH-Xet5 during the resistant potato–PVY^NTN^ interaction. However, during the susceptible interaction there was some decrease in the deposition of XTH-Xet5. Zheng et al. [14] came to a similar conclusion based on the comparative transcriptome analyses of the XTH message. The xyloglucan xylosyltransferase in *Arabidopsis* is potentially expressed in all plant tissues, with a strong presence in roots, stems, but also leaves [37]. In *Arabidopsis*, the immunolocalisation detected this activity in roots, vascular bundles, and in the root epidermis and hairs. Similarly, by using immunolocalisation we demonstrated a strong XTH-Xet5 signal in both vascular tissues and the epidermis in the PVY-infected potato, but during hypersensitive response all leaves showed strong deposition starting 10 days after inoculation. A similar conclusion was made by Antosiewicz et al. [45] based on an abiotic stress stimulation experiment, where the xyloglucan endotransglycosylase was mainly detected in vascular tissues and the epidermis. Like during PVY–potato interaction, the wind stimulated the deposition of XET mainly in the xylem, but also in the mesophyll of *A. thaliana*. XET was also localised in middle lamella or even in the intercellular space [45,46]. 

Ultrastuctural analysis localised XTH-Xet5 in the susceptible potato cultivar in the cell wall and in membranous structures, but also in the ER, trans-Golgi network, and in vacuoles with vesicles. Zabotina et al. [47] also observed high levels of the enzyme in the trans-Golgi network. Accumulation of XTH-Xet5 in membranous structures seems to be logical, considering that the endomembrane complex is known to function as an orchestrated system that delivers the Golgi-derived and endocytic vesicles that carry the cell wall and the cell membrane components [48]. 

Additionally, PVY infection significantly redirected XTH-Xet5 depositions, regardless of the type of interaction, versus the mock-inoculated tissues. The immunogold analysis clearly indicates that the deposition of XTH-Xet5 in resistant and susceptible interactions significantly differed from each other. In the susceptible host XTH-Xet5 dominated in the cell wall and vesicles, whereas in the resistant host it dominated in the cytoplasm, cell wall and trans-Golgi network, generally at a much higher level. Our findings demonstrate that the hypersensitive reaction induces the XTH-Xet5 more actively in the areas of XET synthesis and transports the enzyme more actively to the cytoplasm, cell wall and vacuoles.

## 4. Materials and Methods

### 4.1. Plant Material and Virus Inoculation

Potato plants (*Solanum tuberosum*) of two cultivars with different resistance levels (Irys (PVY^NTN^ resistance score 5.5 in a 1–9 scale) and Sárpo Mira (resistance score 9) [49]) were acquired from IHAR-PIB, Plant Breeding and Acclimatisation Institute, Bonin Research Center, **Bonin, Poland**. Plants were grown and inoculated mechanically as previously presented [11,50] at the four-leaf stage with the NTN strain of PVY. Potato cv. Sárpo Mira developed a hypersensitive necrotic response visible at 7 days post-inoculation. This reaction is conferred by the *Ny-Smira* gene located on the long arm of the potato IX chromosome [51]. Hypersensitive reaction symptoms on inoculated leaves appeared 7 days post-inoculation. Cultivar Irys developed systemic necrosis visible at 10 days post-inoculation. Leaves from both PVY^NTN^-infected plants were collected at three different time intervals to categorise the reaction as susceptible or resistant. In the case of susceptible potatoes (cv. Irys), the leaves were collected 10, 14 and 21 days post-PVY^NTN^ inoculation (dpi), whereas the resistant potato cv. Sárpo Mira leaves were collected after 7, 10 and 14 dpi. Different starting points for collecting the plant material were chosen because of differences in the course of viral infection on either cultivar. Healthy leaves of both cultivars (used as controls) were mock-inoculated with phosphate buffer, at 10 dpi (susceptible) and 7 dpi (resistant). To double-check the potato cultivars, all analysed potato plants were tested for the presence of PVY using ELISA [52]. ELISA testing was performed according to [53], and the results are presented in Table S1. Absorbance was measured at 405 nm. Mean values for ELISA titres were assessed for 50 leaves from each combination (Table S1).

### 4.2. Immunofluorescence Localisation and the Assessment of the Quantitative Fluorescence Signal by Using the Corrected Total Cell Fluorescence Method (CTCF)

Fragments of leaves from PVY^NTN^ and mock-inoculated potato plants (at the abovementioned time intervals) were fixed and embedded in butyl-methyl-methacrylate (BMM) resin according to a procedure described previously [50], with the following modifications. Acetone was used to remove the BMM from 2 μm sections and stuck to silane slides (Thermo-Fisher Scientific, Warsaw, Poland). A further immunofluorescence procedure/analysis was carried out exactly as described in [11]. During analyses we have used two sets of primary and secondary antibodies. For localisation of **XTH-Xet5** the primary rabbit antibodies were acquired from Agrisera (Vӓnӓs, Sweden), whereas the secondary anti-rabbit IgG conjugated with AlexaFluor^®^ 488 was provided by Jackson ImmunoResearch Europe Ltd. (Cambridgeshire, UK). To localise xylan1/xyloglucan, the primary mouse antibodies were obtained from Agrisera (CCRC-M108, recognises glycan group of xylan-1, binds to xylans and to non-fucosylated xyloglucans, Vӓnӓs, Sweden), while the secondary anti-mouse IgG conjugated with AlexaFluor^®^ 488 was from Jackson ImmunoResearch Europe Ltd. The controls consisted of mock-inoculated tissue and a pre-immune serum. An Olympus AX70 Provis (Olympus Poland, Warsaw, Poland) with a UM61002 filter set and an Olympus UC90 HD camera (Olympus Poland) were used for fluorescence imaging. Images were acquired using Olympus Cell Sense Standard Software (Olympus, Center Valley, PA, USA, version 1.18). After gaining florescent images, further quantitative measuring of the fluorescence signal was performed. As a first step, regions (cell wall or protoplast) with green fluorescence signal from xylan-1 or Xet5 epitopes were marked and outlined by using a special marker in the Image J program (Version 1.52e, National Institutes of Health, Bethesda, MD, USA). Next, the measurements of green immunofluorescence signal were done within the outlined cell regions of mock-inoculated plants, and the infected Irys and Sárpo Mira plants with the use of Image J. Levels of fluorescence signal were calculated in the form of corrected total cell fluorescence (CTCF) on magnification 20× with 1.00 zoom factor [54,55] using the following formula:

CTCF = Integrated Density − (Area of Selected Cell Region × Mean Fluorescence of Background Readings)

Estimated CTCF values were then analysed statistically at selected time intervals for both types of reaction to PVY^NTN^ by using the one-factor analysis of variance method (ANOVA). The ANOVA analyses enabled us to find the values of statistical significance when quantifying the levels of Xylan-1 or Xet5. Furthermore, the mean CTCF values were evaluated at the *p* < 0.05 level of significance using post hoc Tukey HSD testing in STATISTICA software (StataSoft and TIBCO Software Inc., Palo Alto, CA, USA, version 13.0).

### 4.3. Quantitative Immunogold Localisation by Direct Estimation of the Relative Labelling Index (RLI)

Potato leaves of both cultivars were fixed and embedded and treated step by step to prepare for transmission electron microscopy (TEM) according to [11]. Then 50–70 nm thick leaf sections from PVY^NTN^-infected or mock-inoculated plants were mounted on Formvar-coated nickel grids and treated exactly as described by Otulak et al. [50]. Grids were rinsed with primary antibodies for XTH (Xet5) or xylan-1/xyloglucan in PBS and washed in PBS-Tween 20. After that the grids with leaf sections were treated for 1 h with gold-conjugated secondary antibody, with anti-rabbit 15 nm (Sigma-Aldrich, Warsaw, Poland) for XTH localisation or with 10 nm for xylan-1/xyloglucan detection (Sigma-Aldrich, Warsaw, Poland), rinsed for 5 min in PBS and then in distilled water. Labelling specificity was checked by incubating grids with material from mock-inoculated plants and by omitting the primary antibody in the incubation solution [11]. The grids were counterstained with 1% uranyl acetate for 5 min and washed 5 × 2 min with distilled water. The immunogold-labelled sections were examined by transmission electron microscope (as described above). The results of immunogold labelling of xylan-1 and Xet5 in mock-inoculated, susceptible or resistant potato plants were further analysed as follows. The quantitative assessment of preferential labelling of specific structures/organelles was carried out by using a reliable estimation method called relative labelling index (RLI). RLI was determined as described by Mayhew [56] and Otulak et al. [57]. The direct estimation method of RLI was selected by comparing the number of observed gold particles (G0) within selected compartments with the expected gold particles (Ge) of the appropriate reference structure or organelles in a leaf [56]. For estimation of G0 and Ge, gold particles were scored in 40 of 10 μm^2^ fields per photo. When there is random labelling, RLI equalled 1, but where there is preferential labelling, RLI was higher than 1. Statistical significance of preferential labelling was assessed by partial *Χ*^2^ analysis according to Mayhew [56]. The statistically significant RLI values were >1, with the corresponding partial *Χ*^2^ values accounted for a significant proportion (at least 10%) of total *Χ*^2^. 

## 5. Conclusions

In this work we have addressed a general question about the function of different cell wall components—particularly, how these elements interact with each other, but also how they change due to interactions with pathogenic viruses. By using precise in situ fluorescence and the ultrastructural localisation of non-cellulosic polysaccharides, we present for the first time the trends of accumulation of the major cell wall matrix hemicelluloses, i.e., xylan-1/xyloglucan together with xyloglucan xyloglucosyl-transferase (XTH-Xet5) in both the symplast and the apoplast during either compatible or incompatible interactions with PVY^NTN^. The function of xyloglucan in the plant cell wall seems to correlate closely with the pathogen-induced changes. Additionally, the loosening of cell wall accompanied the increase in xylan deposition during susceptible Irys–PVY interaction, whereas, during the hypersensitive response, with the cell wall strengthening, the xylan content extensively decreased. Moreover, the level of XTH-Xet5 gradually increases after the resistant potato–PVY^NTN^ interaction; during susceptible interaction the deposition of XTH-Xet5 somewhat decreases. The presented data provide novel insight into the cell wall reorganisation induced by viruses, specifically by PVY^NTN^ infection, illustrating the processes that take place during biotic stress. Our findings increase the understanding of the mechanisms of defence that are actively incorporated into cell wall signalling. 

## Figures and Tables

**Table 1:** Quantification of immunogold labelling by RLI and *Χ*^2^ test of Xyl and XTH. (**A**) Assessment of immunogold labeling of xyl-1/xyloglucan in mock-inoculated and PVY^NTN^ inoculated cv. Irys (susceptible) and cv. Sárpo Mira (resistant) potato plants. (**B**) Assessment of immunogold labelling of XTH-Xet5 in mock-inoculated and PVY^NTN^-inoculated cv. Irys (susceptible) and cv. Sárpo Mira (resistant) potato plants. Significant values (RLI > 1, and % *Χ*^2^ at least 10%) in red bold font and marked with the asterisk (*).

Sample	Parameters of Immunogold Labelling				
	*G_0_*	*Ge*	*RLI*	*Χ* ^2^ *Value*	*Χ* ^2^ *as %*
**(A) Immunogold localisation of xyl-1/xyloglucan:**					
**1. Mock-inoculated cv. Irys potato plants**					
cell wall	27	3	9.00 *	192.00	56.30 *
endoplasmic reticulum (ER)	14	3	4.67 *	40.33	11.83 *
Golgi apparatus	12	2	6.00 *	50.00	14.66 *
chloroplasts	0	6	0.00	6.00	1.76
mitochondrion	1	7	0.14	5.14	1.51
vacuole	1	3	0.33	1.33	0.39
cytoplasm	0	4	0.00	4.00	1.47
vesicles	17	4	4.25 *	42.25	12.39 *
Column total				341.06	100
**2. PVY^NTN^ inoculated cv. Irys potato plants**					
cell wall	77	15	5.13 *	256.27	23.89 *
endoplasmic reticulum (ER)	32	6	5.33 *	112.67	10.50 *
Golgi apparatus	30	4	7.50 *	169.00	15.75 *
chloroplasts	0	5	0.00	5.00	0.47
mitochondrion	0	4	0.00	4.00	0.37
vacuole	64	10	6.40 *	291.60	27.18 *
cytoplasm	1	4	0.25	2.25	0.21
vesicles	74	15	4.93 *	232.07	21.63 *
Column total				1072.85	100
**3. Mock-inoculated cv. Sárpo Mira potato plants**					
cell wall	30	3	10.00 *	243.00	35.69 *
endoplasmic reticulum (ER)	11	2	5.50	40.50	5.95
Golgi apparatus	15	2	7.50 *	84.50	12.41 *
chloroplasts	2	1	2.00	1.00	0.15
mitochondrion	4	3	1.33	0.33	0.05
vacuole	21	2	10.50 *	180.50	26.51 *
cytoplasm	0	3	0.00	3.00	0.44
vesicles	18	2	9.00 *	128.00	18.80 *
Column total				680.83	100
**4. PVY^NTN^ inoculated cv. Sárpo Mira potato plants**					
cell wall	9	2	4.50 *	24.50	20.04 *
endoplasmic reticulum (ER)	7	4	1.75	2.25	1.84
Golgi apparatus	8	2	4.00 *	18.00	14.72 *
chloroplasts	0	3	0.00	3.00	2.45
mitochondrion	0	3	0.00	3.00	2.45
vacuole	7	2	3.50 *	12.50	10.22 *
cytoplasm	12	3	4.00 *	27.00	22.09 *
vesicles	10	2	5.00 *	32.00	26.18 *
Column total				122.25	100
**(B) Immunogold localisation of XTH-Xet5:**					
**1. Mock-inoculated cv. Irys potato plants**					
cell wall	19	3	6.33 *	85.33	25.45 *
endoplasmic reticulum (ER)	22	5	4.40 *	57.80	17.24 *
Golgi apparatus	11	2	5.50 *	40.50	12.08 *
chloroplasts	0	4	0.00	4.00	1.19
mitochondrion	0	4	0.00	4.00	1.19
vacuole	19	3	6.33 *	85.33	25.45 *
cytoplasm	4	2	2.00	2.00	0.60
vesicles	16	3	5.33 *	56.33	16.80 *
Column total				335.30	100
**2. PVY^NTN^ inoculated cv. Irys potato plants**					
cell wall	13	4	3.25 *	20.25	17.62 *
endoplasmic reticulum (ER)	3	2	1.50	0.50	0.44
Golgi apparatus	9	2	4.50 *	24.50	21.32 *
chloroplasts	2	3	0.67	0.33	0.29
mitochondrion	0	3	0.00	3.00	2.61
vacuole	9	2	4.50 *	24.50	21.32 *
cytoplasm	5	3	1.67	1.33	1.16
vesicles	11	2	5.50 *	40.50	35.24 *
Column total				114.92	100
**3. Mock-inoculated cv. Sárpo Mira potato plants**					
cell wall	10	2	5.00 *	32.00	53.93 *
endoplasmic reticulum (ER)	3	2	1.50	0.50	0.84
Golgi apparatus	5	3	1.67	1.33	2.25
chloroplasts	0	3	0.00	3.00	5.06
mitochondrion	0	3	0.00	3.00	5.06
vacuole	7	2	3.50 *	12.50	21.07 *
cytoplasm	0	3	0.00	3.00	5.06
vesicles	3	1	3.00	4.00	6.74
Column total				59.33	100
**4. PVY^NTN^ inoculated cv. Sárpo Mira potato plants**					
cell wall	28	4	7.00 *	144.00	23.80 *
endoplasmic reticulum (ER)	6	2	3.00	8.00	1.32
Golgi apparatus	22	4	5.50 *	81.00	13.38 *
chloroplasts	1	6	0.17	4.17	0.69
mitochondrion	0	6	0.00	6.00	0.99
vacuole	26	4	6.50 *	121.00	19.99 *
cytoplasm	30	4	7.50 *	169.00	27.93 *
vesicles	14	2	7.00 *	72.00	11.90 *
Column total				605.17	100

## Supplementary Materials

The following are available online at http://www.mdpi.com/1422-0067/19/8/2287/s1.
