# Supplementary material for: Spatiotemporal Changes in Xylan-1/Xyloglucan and Xyloglucan Xyloglucosyl Transferase (XTH-Xet5) as a Step-In of Ultrastructural Cell Wall Remodelling in Potato–Potato Virus Y (PVYNTN) Hypersensitive and Susceptible Reaction
Source: Int J Mol Sci. 2018 Aug 4;19(8):2287. doi: 10.3390/ijms19082287 (PMC6121353; doi:10.3390/ijms19082287)
Supplement: Supplementary file 1 [file ijms-19-02287-s001.pdf]

**Supplementary Table S1.** Mean value of ELISA for mock-inoculated and $PVY^{NTN}$ inoculated cv. Irys and cv. Sárpo Mira. Absorbance was measured at 405 nm.

| **Combination** | **Mean value of ELISA titers** |
|---|---|
| Mock-inoculated cv. Irys 10 dpi | 0.010 |
| Mock-inoculated cv. Irys 14 dpi | 0.011 |
| Mock-inoculated cv. Irys 21 dpi | 0.012 |
| $PVY^{NTN}$ inoculated cv. Irys 10 dpi | 1.000 |
| $PVY^{NTN}$ inoculated cv. Irys 14 dpi | 1.320 |
| $PVY^{NTN}$ inoculated cv. Irys 21 dpi | 1.675 |
| Mock-inoculated cv. Sárpo Mira 7 dpi | 0.006 |
| Mock-inoculated cv. Sárpo Mira 10 dpi | 0.004 |
| Mock-inoculated cv. Sárpo Mira 14 dpi | 0.004 |
| $PVY^{NTN}$ inoculated cv. Sárpo Mira 7 dpi | 0.205 |
| $PVY^{NTN}$ inoculated cv. Sárpo Mira 10 dpi | 0.190 |
| $PVY^{NTN}$ inoculated cv. Sárpo Mira 14 dpi | 0.090 |
